# Enhancement of neutrophil autophagy by an IVIG preparation against multidrug-resistant bacteria as well as drug-sensitive strains

**DOI:** 10.1189/jlb.4A0813-422RRR

**Published:** 2015-04-23

**Authors:** Hiroshi Itoh, Hidemasa Matsuo, Naoko Kitamura, Sho Yamamoto, Takeshi Higuchi, Hiromu Takematsu, Yasuhiko Kamikubo, Tadakazu Kondo, Kouhei Yamashita, Masataka Sasada, Akifumi Takaori-Kondo, Souichi Adachi

**Affiliations:** Departments of *Human Health Sciences and ^‡^Hematology and Oncology, Graduate School of Medicine, Kyoto University, Kyoto, Japan; ^†^Department of Clinical Laboratory, Kyoto University Hospital, Kyoto, Japan; and ^§^Department of Hematology and Oncology, Shiga Medical Center for Adults, Shiga, Japan

**Keywords:** xenophagy, bactericidal activity, phagocytosis, NETs

## Abstract

Autophagy occurs in human neutrophils after the phagocytosis of multidrug-resistant bacteria and drug-sensitive strains, including *Escherichia coli* and *Pseudomonas aeruginosa*. The present study detected autophagy by immunoblot analysis of LC3B conversion, by confocal scanning microscopic examination of LC3B aggregate formation and by transmission electron microscopic examination of bacteria-containing autophagosomes. Patients with severe bacterial infections are often treated with IVIG alongside antimicrobial agents. Here, we showed that IVIG induced neutrophil-mediated phagocytosis of multidrug-resistant strains. Compared with untreated neutrophils, neutrophils exposed to IVIG showed increased levels of bacterial cell killing, phagocytosis, O_2_^−^ release, MPO release, and NET formation. IVIG also increased autophagy in these cells. Inhibiting the late phase of autophagy (fusion of lysosomes with autophagosomes) with bafilomycin A1-reduced, neutrophil-mediated bactericidal activity. These findings indicate that autophagy plays a critical role in the bactericidal activity mediated by human neutrophils. Furthermore, the autophagosomes within the neutrophils contained bacteria only and their organelles only, or both bacteria and their organelles, a previously undocumented observation. Taken together, these results suggest that the contents of neutrophil autophagosomes may be derived from specific autophagic systems, which provide the neutrophil with an advantage. Thus, IVIG promotes the neutrophil-mediated killing of multidrug-resistant bacteria as well as drug-sensitive strains.

## Abstract (precis)

IVIG promotes bactericidal activity of neutrophils against multi-drug-resistant bacteria and enhancement of neutrophil autophagy, which may be a critical role in the bactericidal activity of neutrophils.

## Introduction

The overuse and misuse of antibiotics to treat infectious diseases have contributed to the emergence of multidrug-resistant bacteria. In addition, the use of immunosuppressive drugs after organ transplantation, the use of chemotherapeutic agents to treat malignant tumors, and the growing population of elderly patients have increased the number of potential immunocompromised hosts. Infectious diseases, particularly those caused by multidrug-resistant bacteria, are a major cause of death in such immunocompromised individuals; this is because the course of infection is usually severe, and the pathogens are refractory to treatment. Therefore, treatments are focused on enhancing host defenses against microorganisms. Immunocompromised hosts may not be able to synthesize enough Igs to keep up with demand; this is often because many of the antibodies bind to the pathogenic organisms during a severe infection, thereby depleting the general antibody pool.

The combination of IVIG plus antibiotics is an effective treatment for severe infectious diseases [1–4]. Furthermore, IVIG may be administered periodically to immunocompromised patients (such as those receiving immunosuppressive drugs) as a means of preventing infectious diseases. IVIG contains antibodies that are specific for a variety of bacteria or bacterially produced toxins. Although it is thought that the mechanism(s) underlying the antibacterial activity mediated by IVIG involve bacteriolysis and the neutralization of toxins, their ability to increase bacterial susceptibility to antibiotics and/or opsonization (which target bacteria for phagocytosis by neutrophils) have not been fully explored.

Neutrophils are the most common type of white blood cell and play a central role in primary host defenses against bacterial infections. A reduction in the number or function of neutrophils often results in an immunocompromised state, which can lead to critical infections [5, 6]. Many antibiotics are ineffective against drug-resistant bacteria; however, host defenses, including neutrophils, are active against bacteria regardless of whether they are drug sensitive or resistant. Thus, the improved treatment outcomes observed for patients with severe infections that are treated with antibiotics and IVIG [1, 2] may be a result of the increased bactericidal activity of neutrophils.

Here, we used IgG-free serum supplemented with complement proteins to mimic the environment within an immunocompromised host and examined the effect of IVIG on neutrophil-mediated bactericidal activity against multidrug-resistant strains of *E. coli* and *P. aeruginosa* (both of which cause severe nosocomial infections). The effects of IVIG on neutrophil bactericidal function were assessed by measuring the phagocytosis index, the production of ROS, MPO activity, and NET formation.

Autophagy is an essential homeostatic process, in which cells degrade their own (often damaged) components [7, 8]. The process can occur via 3 major pathways: macroautophagy, microautophagy, or chaperone-mediated autophagy. Interestingly, stimulating neutrophils with *E. coli*, which is not an intracellular parasite, induce autophagy [9], suggesting that the autophagic machinery may contribute to the bactericidal activity of neutrophils. Accumulating evidence suggests that the autophagic machinery is also involved in the clearance of pathogenic bacteria [10, 11], a process termed xenophagy. Therefore, we examined the effect of IVIG on macroautophagy and xenophagy in neutrophils stimulated with drug-resistant bacteria. Moreover, we considered the contribution of the LAP pathway [12] to this process.

## MATERIALS AND METHODS

### Reagents

Dulbecco’s PBS(−), heart-infusion broth, and trypticase soy broth were purchased from Nissui Pharmaceutical (Tokyo, Japan). HBSS supplemented with Ca^2+^ and Mg^2+^ was purchased from Gibco Invitrogen (Life Technologies, Carlsbad, CA, USA). Gentamycin sulfate, trisodium citrate dihydrate, dextran (MW ∼200,000 Da), paraformaldehyde, glutaraldehyde, OsO_4_, and RIPA buffer containing a protease inhibitor cocktail were purchased from Nacalai Tesque (Kyoto, Japan). Cytochrome c, apocynin, DPI, sheep serum, and zymosan were purchased from Sigma-Aldrich (St. Louis, MO, USA). Bafilomycin A1, glycine, SDS, 2-amino-2-hydroxymethyl-1,3-propanediol [Tris (hydroxymethyl) aminomethane], SOD, and polyoxyethylene [10] octylphenyl ether (Triton X-100) were purchased from Wako Pure Chemical Industries (Osaka, Japan). DNase was purchased from Roche (Basel, Switzerland).

### IgG-free serum

Human blood type AB serum was used as a source of complement. To ensure that the effects examined were specific to IVIG, IgG was first removed from the serum by protein G affinity chromatography. The serum was provided from Kaketsuken (Kumamoto, Japan). No IgG was detectable in the serum; however, it did contain IgA (0.74 mg/ml), IgM (0.39 mg/ml), and complement components C3 (0.54 mg/ml) and C4 (0.15 mg/ml) and had a CH50 value of 19.6 U/ml. Furthermore, endotoxin was present in the serum at a concentration of 0.068 endotoxin units/ml, suggesting that the serum was not contaminated with bacteria.

Treated serum was stored at −80°C until use. The concentration of IgG-free serum used in the present study was low (as little as 1%) to ensure that the effects of IVIG were being measured.

### IVIG

Kenketsu Venilon-I (dialyzed against PBS) was used as the source of IVIG (Teijin Pharma, Tokyo, Japan). Previous reports show that administering 5 g IVIG to agammaglobulinemic patients raises the serum IgG concentration to ∼2 mg/ml, even when the IVIG is administered in accordance with the Japanese guidelines for the administration of IVIG in cases of severe infection (5 g/d for 3 days). Therefore, serum IgG levels ≥2 mg/ml were anticipated in the present study, which used IVIG preparations at a concentration of 1 mg/ml (which is consistent with the concentration range found in IgG preparations derived from patient sera). Kenketsu albumin (Kaketsuken) was used as a control.

### Bacterial strains

Drug-sensitive strains of *E. coli* and *P. aeruginosa*, ESBL-producing strains of *E. coli*, and MDRP were isolated from patients in Kyoto University Hospital (Japan). Identification of the bacteria and antibiotic susceptibility tests was carried out according to the methods recommended by the Clinical and Laboratory Standards Institute (Wayne, PA, USA). The results of the antibiotic susceptibility tests were described in Supplemental Table 1. The strains were cultured in heart-infusion broth and preserved at −80°C in stock medium (KEEP MEDIUM; Nikken Biomedical Laboratory, Kyoto, Japan). Before use, the strains were incubated overnight in trypticase soy broth, washed 3 times in PBS, and then suspended in HBSS. The bacterial concentration was adjusted by measuring the absorbance of the bacterial suspension at 600 nm. At least 3 strains of drug-sensitive or multidrug-resistant bacteria were examined once each.

### Neutrophil preparation

Citrated venous blood was obtained from healthy adult volunteers and neutrophils isolated by dextran sedimentation followed by gradient centrifugation on a 2-step Percoll gradient (GE Healthcare Bio-Sciences, Uppsala, Sweden), as described previously [13].

All volunteers provided written, informed consent, and the protocol was approved by the Institutional Review Board of Kyoto University Hospital.

### Bactericidal assay (bacterial viability in whole samples)

A reaction mixture containing 1% IgG-free serum, bacteria (2.5 × 10^6^ cfu/ml), human neutrophils (2.5 × 10^6^ cells/ml), and 1 mg/ml IVIG (total volume = 0.5 ml) was incubated for 60 minutes or 120 minutes at 37°C with gentle shaking. A second reaction mixture was also prepared, which did not contain neutrophils. After incubation, the reaction mixtures were diluted in excess sterile, distilled water and inoculated onto heart-infusion agar plates. After culturing for 24–48 hours, the number of colonies on each plate was counted.

### Bactericidal assay (viability of bacteria inside of neutrophils)

Neutrophils (2.0 × 10^6^ cells/ml) and bacteria (1.0 × 10^8^ cfu/ml) were mixed in 0.5 ml HBSS containing 10% autologous serum, with or without the autophagy inhibitor bafilomycin A1 (500 nM), the NADPH oxidase inhibitor apocynin (300 *µ*M), or DPI (1 *µ*M) for 90 minutes at 37°C with gentle shaking. Then, to kill the nonengulfed extracellular bacteria, the cell membrane-impermeable antibiotic gentamicin was added at a final concentration of 100 *µ*g/ml for 30 minutes. After the neutrophil pellets were washed with PBS (washing reduced the gentamicin concentration in the reaction tube to <100 ng/ml), the pellets were resuspended in 1 ml sterile, distilled water and left for 30 minutes at 4°C. The sample was then diluted in sterile, distilled water and inoculated onto heart-infusion agar plates. After culturing for 24 hours, the number of colonies on each plate was counted.

### Phagocytosis assay

Neutrophils (2.5 × 10^6^ cells/ml) and bacteria (2.5 × 10^7^ cfu/ml) were mixed in 0.5 ml HBSS containing 1% IgG-free serum and then incubated with or without 1 mg/ml IVIG for 30 minutes at 37°C with gentle shaking. After incubation, the samples were centrifuged at 2300 *g* for 2 minutes. The neutrophil pellet was washed with PBS and then treated with PBS containing 0.1% Trypsin and 0.02% EDTA (Dainippon Sumitomo Pharma, Osaka, Japan) for 15 minutes at room temperature to remove any bacteria attached to the cell membrane that had not been engulfed. Neutrophils were resuspended in PBS containing 10% human AB serum. Cytospin specimens were prepared by use of a Cytofuge 2 cytocentrifuge (265 *g* for 2 minutes; StatSpin Technologies, Norwood, MA, USA). The cytospin specimens were then stained with Diff-Quik (Sysmex, Kobe, Hyogo, Japan) and examined under a microscope. The number of bacteria engulfed by 100 randomly selected neutrophils was counted. The phagocytic activity was measured, according to the rate of phagocytosis and the phagocytosis index. The rate of phagocytosis was calculated as the percentage of neutrophils in the sample that contained more bacteria than those in the time 0 sample. The phagocytosis index was calculated as the average number of bacteria/neutrophils (counting only neutrophils that contained bacteria).

### O_2_^−^release assay

Reaction mixtures (0.5 ml), containing 80 *µ*M cytochrome c, neutrophils (2.5 × 10^6^ cells/ml), bacteria (2.5 × 10^7^ cfu/ml), and 1% IgG-free serum (with or without 1 mg/ml IVIG), were incubated for 30 minutes at 37°C. The reaction mixtures were then centrifuged at 1000 *g* for 10 minutes at 4°C and the supernatants collected. The amount of O_2_^−^ released by the neutrophils was determined by measuring the change in absorbance in the presence and absence of SOD (150 U/ml; absorbance in the absence of SOD − absorbance in the presence of SOD) at 550 nm. The results were expressed as the number of nanomoles of O_2_^−^/1.25 × 10^6^ cells (absorption coefficient, 2.1 × 10^4^ M^−1^ cm^−1^). Data were expressed as the means ± se of duplicate measurements.

### MPO release assay

Neutrophils (2.5 × 10^6^ cells/ml) were incubated for 30 minutes with 1 mg/ml IVIG, with or without bacteria (2.5 × 10^6^ cfu/ml), in 0.5 ml HBSS containing 1% IgG-free serum. Supernatants were collected after centrifugation at 1500 *g* for 5 minutes at 4°C and then passed through a 0.2 *µ*m filter. Samples were stored at −80°C until use.

The EnzChek MPO Activity Assay Kit (Molecular Probes, Life Technologies) was used for the rapid and sensitive determination of MPO activity, according to the manufacturer's instructions. Fluorescence was measured by use of a fluorescence microplate reader (Infinite M200; Tecan, Männedorf, Switzerland) at excitation and emission wavelengths of 485 nm and 530 nm, respectively. Background fluorescence, measured in each of the 0 MPO control reactions, was subtracted from each sample reading before the results were plotted.

### Scanning electron microscopic analysis of NET formation

Reaction mixtures (0.5 ml) containing neutrophils (2.5 × 10^6^ cells/ml), bacteria (2.5 × 10^7^ cfu/ml), 1% IgG-free serum (with or without 1 mg/ml IVIG), and 10 *µ*g/ml DNase were incubated in 4-well glass-bottom chamber slides (Lab-Tek 177399; Thomas Scientific, Swedesboro, NJ, USA) in a humidified incubator (5% CO_2_) for 120 minutes at 37°C. The samples were then centrifuged at 200 *g* for 5 minutes and the supernatants removed. The cell pellets were fixed with 2% glutaraldehyde, postfixed with 1% OsO_4_/1% tannic acid, dehydrated through a graded ethanol series, and then immersed in *t*-butylalcohol. The sample was dried in a freeze dryer (ES-2030; Hitachi, Tokyo, Japan), attached to a base, coated with platinum-palladium alloy (Eiko IB3, Tokyo, Japan), and observed by use of an S-4700 SEM (Hitachi).

### Immunoblot analysis

Neutrophils (∼5 × 10^6^) were washed with PBS and then lysed in RIPA buffer containing a protease inhibitor cocktail. After centrifugation, the protein content of the supernatants was measured by use of the DC Protein Assay (Bio-Rad Laboratories, Hercules, CA, USA). Equal amounts of whole-cell lysate were separated on a 15% SDS polyacrylamide gel and then transferred to a polyvinylidene difluoride membrane (GE Healthcare Bio-Sciences). The membrane was blocked with 0.3% skim milk/TBST for 1 hour, followed by incubation overnight at 4°C with an anti-LC3B polyclonal antibody (1/500; L7543; Sigma-Aldrich) or an anti-GAPDH mAb (1/200; SC47724; Santa Cruz Biotechnology, Santa Cruz, CA, USA). After washing thoroughly in TBST, the membrane was incubated with HRP-conjugated whole anti-rabbit Ig (NIF824; GE Healthcare Bio-Sciences) or anti-mouse Ig (NIF825; GE Healthcare Bio-Sciences), respectively (1/5000), for 1 hour at room temperature. Immunoreactive proteins were detected by use of the HRP Novex ECL Chemiluminescent Substrate Regent Kit (Invitrogen). Signals were captured, and the intensity of the bands was quantified by use of the ChemiDoc XRS System (Bio-Rad Laboratories).

### Confocal immunofluorescence microscopy

Neutrophils (2.5 × 10^6^/ml) were incubated with or without bacteria and IVIG in the presence of 1% IgG-free serum for 90 minutes. Following centrifugation, the cells were resuspended in PBS containing 10% autologous serum and spun onto a glass slide in a Cytofuge 2 cytocentrifuge (140 *g* for 2 min). The cells were then fixed in 4% paraformaldehyde for a minimum of 15 minutes at 4°C and then washed 3 times with PBS. Permeabilization and blocking were performed in buffer A (PBS containing 10% sheep serum and 0.2% Triton X-100) for 1 hour at room temperature. For indirect immunostaining, the cells were incubated in a humid chamber at 4°C overnight with an anti-LC3B mAb (1/100; SAB4200361; Sigma-Aldrich) diluted in buffer A. After washing 3 times in PBS, the cells were incubated with Cy3-conjugated sheep anti-mouse IgG (1/20; C2181; Sigma-Aldrich), followed by a FITC-conjugated anti-*E. coli* polyclonal antibody (1/20; PA1-73029; Thermo Fisher Scientific K. K., Yokohama, Kanagawa, Japan; diluted in buffer A) for 2 hours at room temperature in a dark, humid chamber. In LC3B and ATG5 double-immunostaining, *E. coli* was stained by DAPI, not by FITC-conjugated anti-*E. coli* polyclonal antibody, and ATG5 was stained by anti-ATG5 polyclonal antibody (1/200; PAB0712; Abnova, Zhongli District, Taoyuan City, Taiwan), diluted in buffer A and FITC-conjugated sheep anti-rabbit IgG (1/100; F7512; Sigma-Aldrich). The cells were then washed 3 times with PBS, counterstained with DAPI, and mounted on glass slides by use of ProLong Gold Antifade Reagents (Life Technologies). Slides were analyzed under a confocal laser-scanning microscope (FluoView FV10i; Olympus, Tokyo, Japan), and the images were processed by use of FV10-ASW viewer (Version 03.00.03.00; Olympus).

### TEM

Neutrophils (2.5 × 10^6^) were collected by centrifugation, fixed in 0.1 M phosphate buffer containing 4% paraformaldehyde/2% glutaraldehyde at 4°C, washed in isotonic phosphate-buffered sucrose, and then refixed in phosphate-buffered 1% OsO_4_. Following dehydration in a graded series of ethanol washes, the cells were embedded in Luveak-812 (Nacalai Tesque). Thin sections (70–90 nm thick) were cut on an EM UC6 ultramicrotome by use of a diamond knife (Leica, Heidelberg, Germany), stained with uranyl acetate and lead citrate and then observed under an H-7650 electron microscope (Hitachi).

### Statistical analysis

Data were analyzed by use of the Student’s paired *t*-test. Data were expressed as the mean ± se of at least 3 independent bacterial strains/experiment. Significance levels are indicated in the figure legends.

## RESULTS

### IVIG promotes the bactericidal activity of neutrophils against drug-resistant bacteria

**Figure 1.**
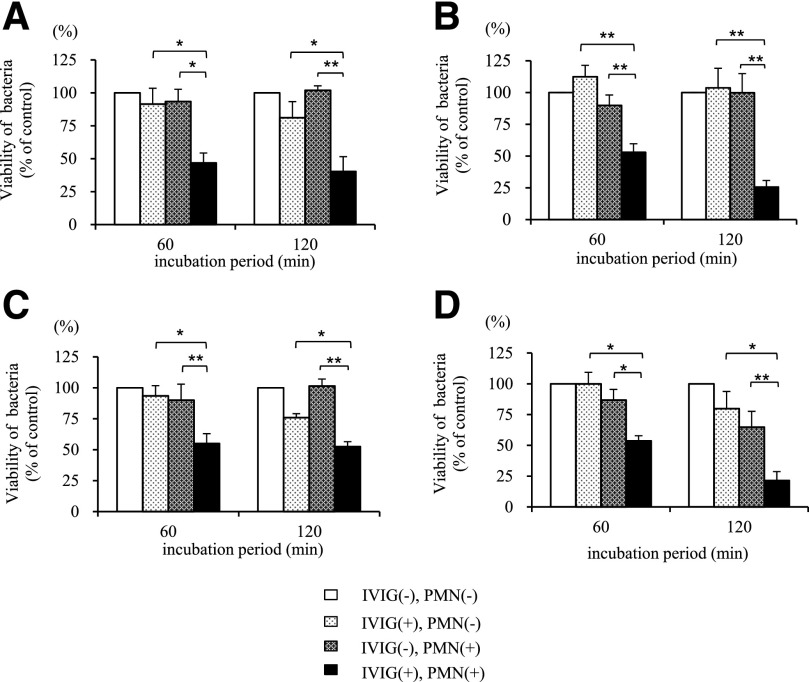
Effect of IVIG on neutrophil-mediated bactericidal activity against multidrug-resistant bacteria. The effect of IVIG on neutrophil-mediated bactericidal activity against drug-resistant strains of *E. coli* (A; *n* = 4 independent strains) and *P. aeruginosa* (B; *n* = 7 independent strains) and against drug-sensitive strains of *E. coli* (C; *n* = 4 independent strains) and *P. aeruginosa* (D; *n* = 4 independent strains). The number of viable bacteria remaining after incubation with 1% IgG-free serum (in the presence or absence of human neutrophils) plus 1 mg/ml IVIG for the indicated times. Data are expressed as a percentage of the value in the control sample (no neutrophils or IVIG). Values represent the means ± se. *P < 0.05; **P < 0.01. PMN, Polymorphonuclear leukocyte.

First, we compared the effects of IVIG on the bactericidal activity of isolated human neutrophils against multidrug-resistant bacteria, drug-resistant strains of *E. coli* (**Fig. 1A**) and *P. aeruginosa* (Fig. 1B), and drug-sensitive strains of *E. coli* (Fig. 1C) and *P. aeruginosa* (Fig. 1D). In the presence of neutrophils, IVIG treatment led to a marked and time-dependent reduction in the number of viable bacteria. In the absence of neutrophils, IVIG treatment led to a slight reduction in the number of viable bacteria (the exception was drug-resistant *P. aeruginosa*). No neutrophil-mediated killing of bacteria occurred in the absence of IVIG.

### IVIG promotes phagocytosis of drug-resistant bacteria by neutrophils

**Figure 2.**
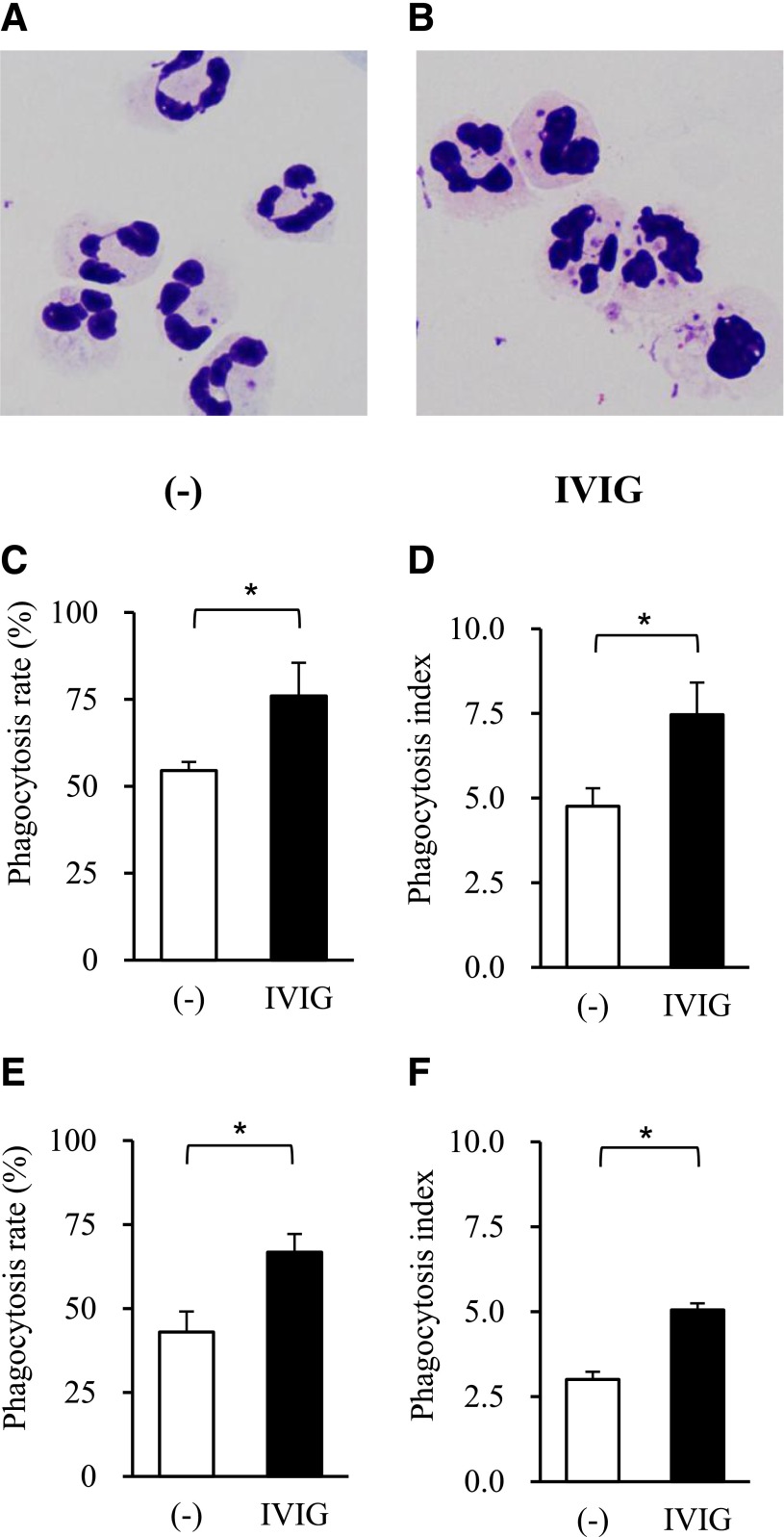
Phagocytosis of multidrug-resistant bacteria by human neutrophils. Phagocytosis of drug-resistant *E. coli* strains by human neutrophils in the absence (A) or presence (B) of IVIG. Original magnification, ×1000. The phagocytosis rate (%; C and E) and the phagocytosis index (D and F) of drug-resistant *E. coli* (C and D; *n* = 4 independent strains) and *P. aeruginosa* (E and F; *n* = 4 independent strains). Data represent the means ± se. *P < 0.05.

A phagocytosis assay, in which neutrophils were cocultured with bacteria at a ratio of 1:10 for 30 minutes in the presence of IgG-free serum, was performed to examine whether IVIG had any effect on neutrophil function (**Fig. 2A–F**). Microscopic examination revealed that phagocytosis of drug-resistant *E. coli* increased markedly in the presence of IVIG (Fig. 2A and B). Phagocytic function was semiquantified by measuring the phagocytic rate and the phagocytic index (Fig. 2C and D, *E. coli*; Fig. 2E and F, *P. aeruginosa*). Samples at time 0 (background controls) represented bacteria that were adhered to the neutrophil membrane but were not engulfed or removed by trypsin treatment. The phagocytosis rate and phagocytic index for drug-resistant strains of *E. coli* and *P. aeruginosa* increased significantly in the presence of IVIG (*P* < 0.05).

### IVIG promotes O_2_^−^release from neutrophils stimulated by drug-resistant bacteria

**Figure 3.**
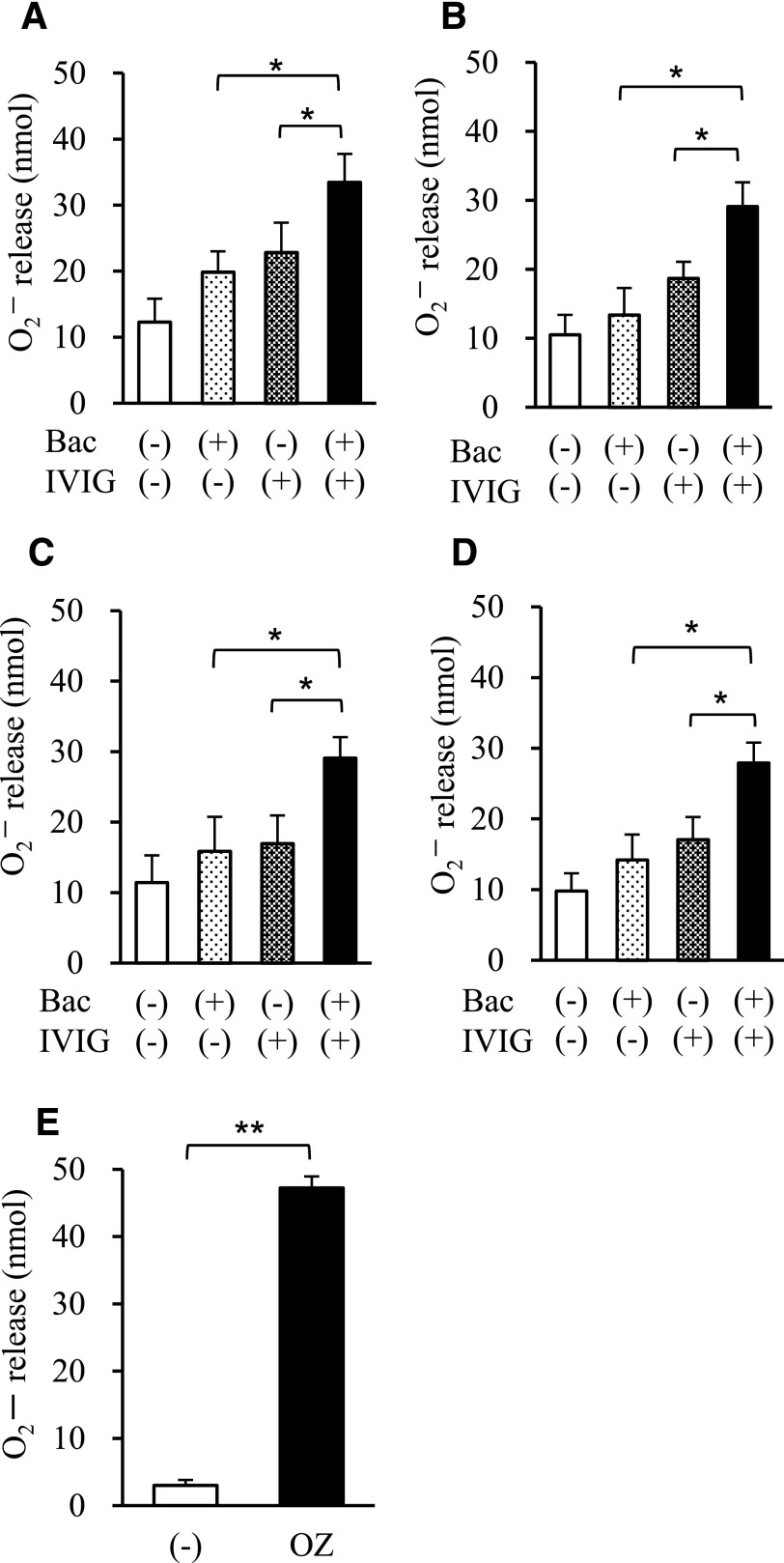
Effect of IVIG on O_2_^−^ release by human neutrophils. The effect of IVIG on O_2_^−^ release by human neutrophils in the presence of 1% IgG-free serum in the presence or absence of 1 mg/ml IVIG, drug-resistant *E. coli* (A; *n* = 4 independent strains) and *P. aeruginosa* (B; *n* = 4 independent strains), or drug-sensitive strains of *E. coli* (C; *n* = 4 independent strains) and *P. aeruginosa* (D; *n* = 4 independent strains). (E) Positive control [1 mg/ml opsonized zymosan (OZ); *n* = 6]. Data represent the means ± se. *P < 0.05; **P < 0.01. Bac, bacteria.

To investigate the effects of IVIG on the production and release of bactericidal factors by neutrophils, we next examined the cellular release of the ROS, O_2_^−^, which is one of the most powerful bactericidal factors produced following the stimulation of phagocytosis [14]. As shown in **Fig. 3**, IVIG induced a marked increase in the amount of O_2_^−^ released in the presence of bacteria (Fig. 3A–D). Importantly, a similar result was noted when neutrophils were stimulated with drug-sensitive or drug-resistant *E. coli* or *P. aeruginosa*. When we examined the dose dependency of IVIG preparations on O_2_^−^ release stimulated by drug-resistant strains of *E. coli*, the amount of O_2_^−^ release increased in a dose-dependent manner (from 0.1 to 3 mg/ml IVIG) but decreased at doses of >10 mg/ml IVIG (data not shown). These data are consistent with the results of a previous study showing that low IVIG concentrations (1–5 mg/ml) induce neutrophil activation but that higher concentrations actually inhibit activation [15].

### IVIG promotes MPO release and extracellular trap formation by neutrophils stimulated by drug-resistant bacteria

**Figure 4.**
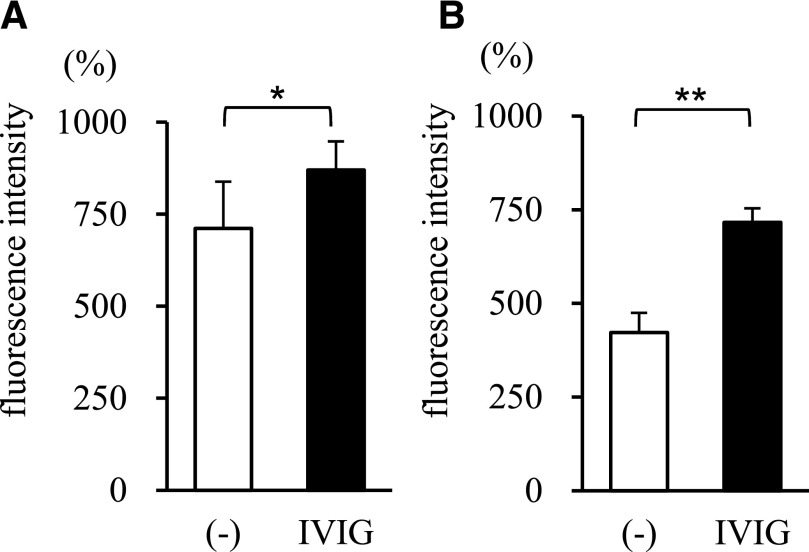
Effect of IVIG on MPO release by human neutrophils. The effect of IVIG on MPO release by human neutrophils in the presence of drug-resistant *E. coli* (A; *n* = 5 independent strains) or *P. aeruginosa* (B; *n* = 4 independent strains). MPO activity is expressed in terms of fluorescence intensity, which was measured after the incubation of neutrophils with 1% IgG-free serum, with or without 1 mg/ml IVIG, and is expressed as a percentage of that in the time 0 sample. Data represent the means ± se. *P < 0.05; **P < 0.01.

The IVIG-induced increase in neutrophil bactericidal activity was confirmed by measuring MPO activity. **Figure 4** shows that MPO release by neutrophils was significantly greater in the presence of bacteria than in the absence of bacteria (Fig. 4A, drug-resistant *E. coli*; Fig. 4B, drug-resistant *P. aeruginosa*). Thus, neutrophils release MPO when they phagocytose bacteria. The amount of MPO released by neutrophils stimulated by drug-resistant strains of *E. coli* (Fig. 4A) or *P. aeruginosa* (Fig. 4B) increased significantly in the presence of IVIG.

We next examined the formation of NETs by SEM. IVIG induced a significant increase in NET formation (NETs trap and kill extracellular bacteria) [16] by neutrophils in response to multidrug-resistant strains of *E. coli* (Supplemental Fig.1). The lack of these mesh-like structures in DNase-treated samples confirmed that they were indeed NETs.

### IVIG promotes autophagy in neutrophils stimulated with drug-resistant bacteria

**Figure 5.**
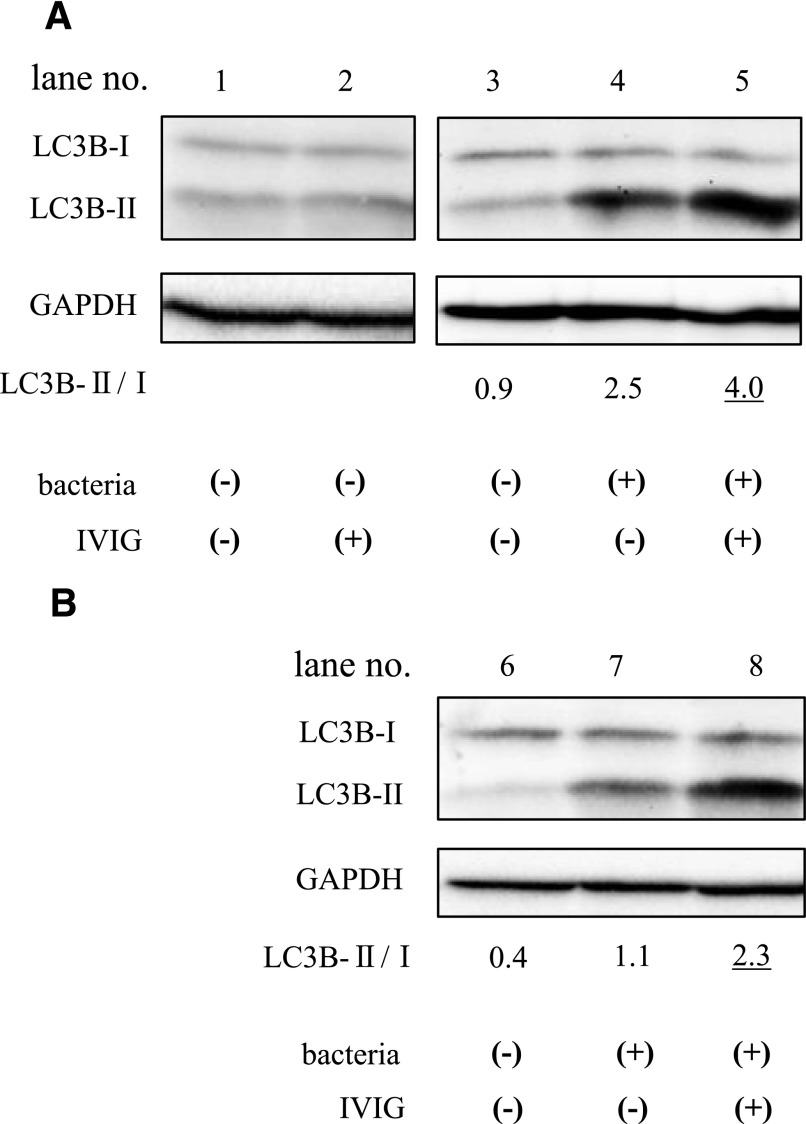
Analysis of LC3B conversion in human neutrophils stimulated with drug-resistant bacteria. Neutrophils were incubated for 90 minutes with or without bacteria (A, drug-resistant *E. coli*; B, drug-resistant *P. aeruginosa*) and IVIG in the presence of 1% IgG-free serum. The samples were then subjected to immunoblot analysis for LC3B (18 and 16 kDa bands), a marker of autophagy. GAPDH (37 kDa) was used as the control. Data are representative of 3 independent experiments by use of different bacterial strains, respectively.

To investigate the effects of IVIG on the induction of neutrophil autophagy in the presence of drug-resistant bacterial strains (**Fig. 5A**, *E. coli*; Fig. 5B, *P. aeruginosa*), neutrophils were incubated in the presence of 1% IgG-free serum, with or without bacteria and/or IVIG. Autophagy was measured by examining the conversion of LC3B-I to LC3B-II by immunoblot analysis. In the absence of bacteria and IVIG, LC3B-I was predominant (Fig. 5A, lanes 1 and 3, and B, lane 6). These results suggest that there was little or no induction of autophagy. In the presence of bacteria but in the absence of IVIG, LC3B-II levels were slightly higher than those of LC3B-I (*E. coli*, Fig. 5A, lane 4; *P. aeruginosa,* Fig. 5B, lane 7). In the presence of *E. coli* (Fig. 5A, lane 5) or *P. aeruginosa* (Fig. 5B, lane 8) and IVIG, LC3B-II was the dominant form of LC3B. These results indicate that IVIG increases the level of autophagy in bacteria-stimulated neutrophils.

**Figure 6.**
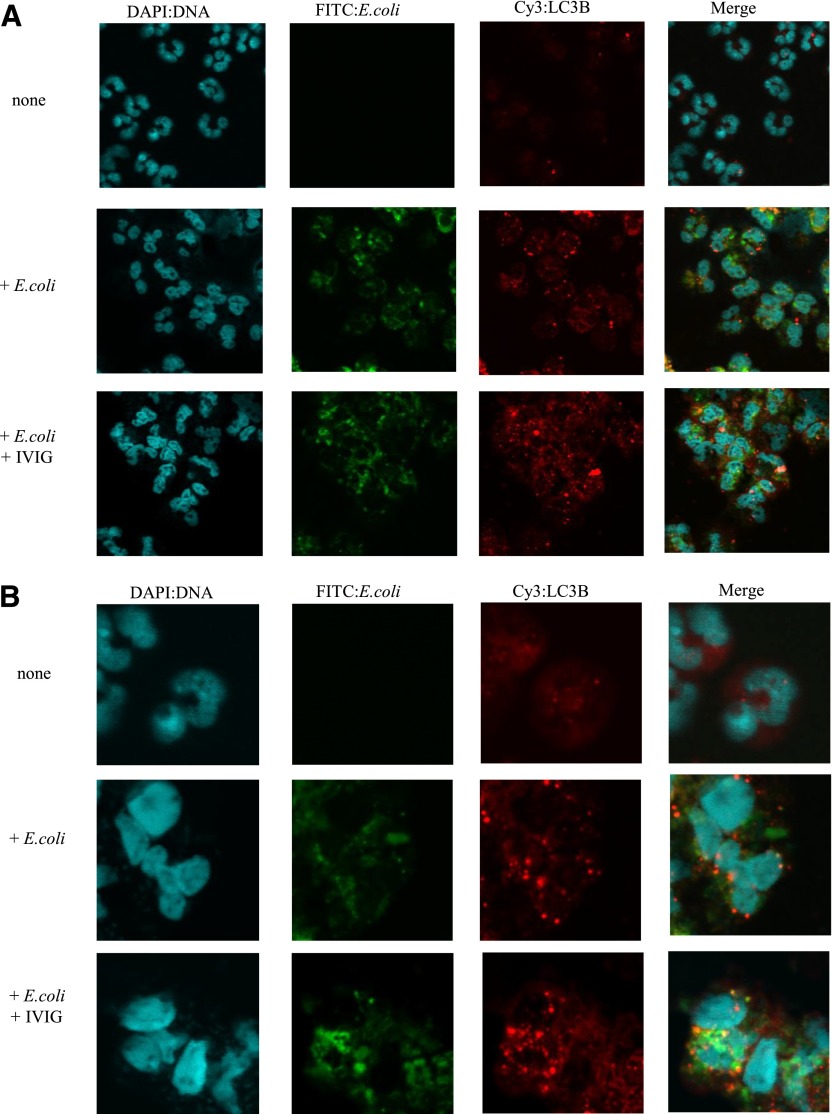

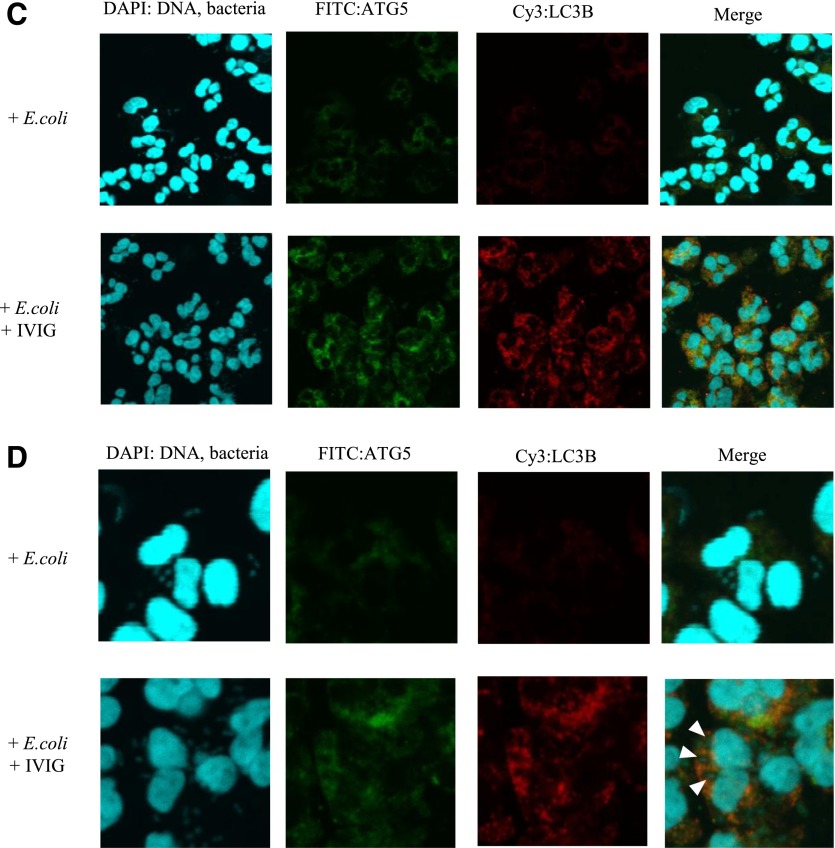
Formation of LC3B aggregates in the cytoplasm of human neutrophils stimulated with drug-resistant *E. coli*. Formation of LC3B aggregates was examined under a confocal laser-scanning microscope. Neutrophils were incubated with (+*E. coli*) or without (none) bacteria (A, ×200 magnification; B, ×600 magnification) and IVIG in the presence of 1% IgG-free serum for 90 minutes. The cells were then examined after indirect immunostaining. The cell nuclei are blue (DAPI), *E*. *coli* are green (FITC), and LC3B is red (Cy3). Indirect double-immunostaining of LC3B and ATG5 was also performed. Neutrophils were incubated with (*+E. coli*, +IVIG) or without (+*E. coli*) IVIG in the presence of 1% IgG-free serum and bacteria for 90 minutes (C, ×200 magnification; D, ×600 magnification).The cell nuclei and bacteria are blue (DAPI), ATG5 are green (FITC), and LC3B is red (Cy3). (D) The arrowheads indicate that LC3B and Atg5 aggregated on the membranes surrounding the bacteria. Data are representative of 3 independent experiments by use of different bacterial strains, respectively.

To investigate the increase in autophagy mediated by IVIG in more detail, we examined the formation of autophagosomes by measuring LC3B aggregation in the cytoplasm of bacteria-stimulated neutrophils. In the absence of bacteria, LC3B aggregates were barely detectable (**Fig. 6A and B**; “none”). In the presence of bacteria, LC3B aggregates and bacteria were observed in the cytoplasm (Fig. 6A and B; “*+E. coli*”). There was a marked increase in LC3B aggregate formation and the number of bacteria present in the neutrophil cytoplasm in the presence of bacteria plus IVIG (Fig. 6A and B; “+*E. coli*, *+*IVIG”). These results showed that IVIG increases the formation of autophagosomes in the cytoplasm of bacteria-simulated neutrophils. We also confirmed by indirect double-immunostaining of LC3B and other important marker of autophagosomes, ATG5. In the presence of bacteria, the Atg5 signal increased after treatment with IVIG, the same as LC3B, and aggregated on the membranes surrounding the bacteria in the cytoplasm (Fig. 6C and D; “+*E. coli*, *+*IVIG”).

**Figure 7.**
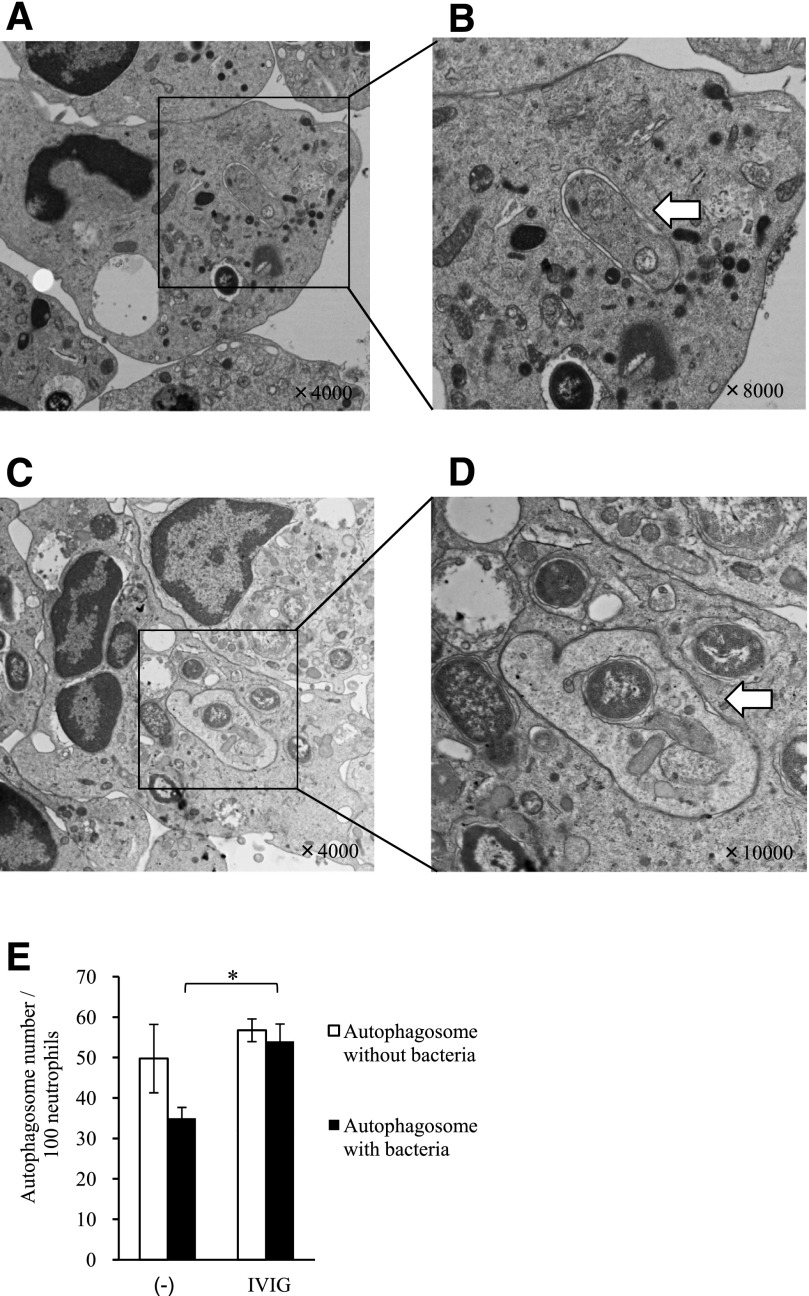
Analysis of autophagy in human neutrophils stimulated with drug-resistant *E. coli*. Neutrophils were incubated with drug-resistant *E. coli*, without or with IVIG in the presence of 1% IgG-free serum for 90 minutes. The samples were then examined by TEM. (A and B) The magnified images are representative of the entire sample. (C) Organelles contained within autophagosomes. (D) The arrow indicates bacteria contained within autophagosomes. The formation of a double-membrane is characteristic of autophagosomes but not phagosomes. Data are representative of 4 independent experiments by use of different bacterial strains. (E) The number of organelles contained within autophagosomes (= total number of typical autophagosomes and autophagosome-like vesicles) and the number of bacteria contained within autophagosomes in 100 cells were determined for each sample (*n* = 4 independent strains). An autophagosome-like vesicle is an autophagosome in which the characteristic double-membrane structure is unclear as a result of the digestion process. Data represent the means ± se. *P < 0.05.

The induction of autophagy was confirmed by TEM. In the absence of IVIG, very few bacteria were phagocytosed by neutrophils (data not shown). By contrast, numerous bacteria were observed in the cytoplasm of neutrophils in the presence of IVIG (**Fig. 7C**). Interestingly, bacteria contained within autophagosomes, which are distinguishable by their characteristic double-membrane, were also observed in the cytoplasm of the neutrophils (Fig. 7D, arrow). Importantly, autophagosomes containing neutrophil organelles, rather than bacteria, were also observed in neutrophils under the same conditions (Fig. 7B, arrow). Thus, it appears that macroautophagy and xenophagy were induced simultaneously in a single neutrophil. We next counted the number of autophagosomes contained within 100 neutrophils in the presence or absence of IVIG and found no significant difference in the number of autophagosomes that contained no bacteria; however, the number of autophagosomes that did contain bacteria was significantly higher in the presence of IVIG (Fig. 7E).

### Inhibiting the late phase of autophagy with bafilomycin A1 reduces the bactericidal activity of neutrophils stimulated by drug-resistant *E. coli*

To investigate the relationship between the bactericidal activity and autophagy in neutrophils, we next examined the effect of an autophagy inhibitor bafilomycin A1, which inhibits the fusion of autophagosomes with lysosomes, on the bactericidal activity of neutrophils. The NADPH oxidase inhibitor, apocynin, and DPI (which blocks O_2_^−^ release) were also used. As the replication of nonengulfed extracellular bacteria interferes with the evaluation of bactericidal activity by neutrophils, the bactericidal assay was performed in the presence of gentamicin to ensure that we evaluated only the intracellular bacteria ingested by neutrophils (as described in Materials and Methods).

**Figure 8.**
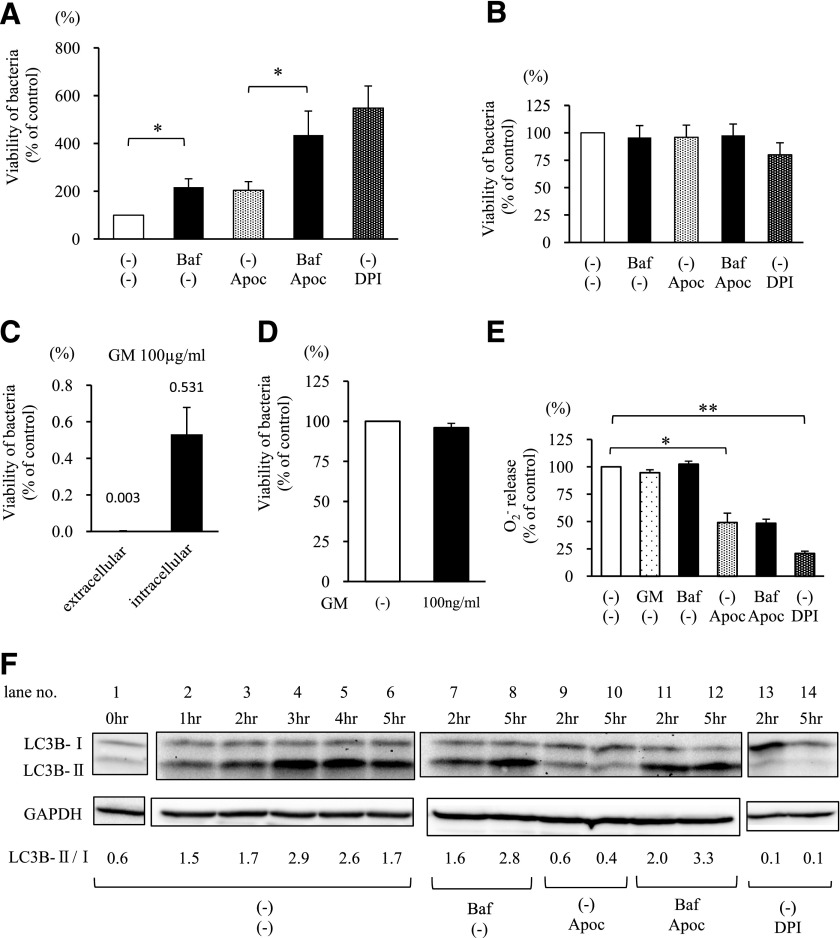
Effects of an autophagy inhibitor, bafilomycin A1, on bactericidal activity of human neutrophils against drug-resistant *E. coli*. (A) Bacterial viability test for intracellular drug-resistant *E. coli* within neutrophils (*n* = 5 independent strains). (B) Effect of bafilomycin A1 (Baf) and NADPH oxidase inhibitors, apocynin (Apoc), and DPI, on the viability of drug-resistant *E. coli* (*n* = 5 independent strains). Bacteria (1.0 × 10^8^ cfu/ml) were incubated for 120 minutes at 37°C in the presence of 10% autologous serum, with or without the inhibitors. (C and D) Bactericidal effect of gentamicin against drug-resistant *E. coli* (*n* = 5 independent strains). Bacteria (1.0 × 10^8^ cfu/ml) were incubated for 90 minutes at 37°C in the presence of 10% autologous serum. Then, gentamicin [GM; final concentration of 100 *µ*g/ml (C; “extracellular”) or 100 ng/ml (D; black bar) or HBSS (D; white bar)] was added for a further 30 minutes. (C) “intracellular,” Percentage of bacteria inside of the neutrophils. (E) Effect of the reagents used in this assay (gentamicin, bafilomycin A1, apocynin, and DPI) on O_2_^−^ release by human neutrophils in the presence of drug-resistant *E. coli* (*n* = 5 independent strains). Neutrophils (2.5 × 10^6^ cells/ml) were pretreated with or without the autophagy inhibitor bafilomycin A1 (500 nM) and the NADPH oxidase inhibitors apocynin (300 *µ*M) or DPI (1 *µ*M) for 10 minutes at 37°C in the presence of 1% IgG-free serum and 1 mg/ml IVIG. Next, bacteria (2.5 × 10^7^ cfu/ml) were added and incubated for a further 30 minutes at 37°C with gentle shaking. (F) Effect of bafilomycin A1 and NADPH oxidase inhibitors on LC3B conversion in human neutrophils stimulated with drug-resistant bacteria. Neutrophils were incubated at 37°C for the indicated times, with or without bafilomycin A1, apocynin, or DPI, in the presence of drug-resistant *E. coli*, 1% IgG-free serum, and 1 mg/ml IVIG. Following incubation, the samples were subjected to immunoblot analysis for LC3B and GAPDH. Data are representative of 3 independent experiments by use of different bacterial strains. *P < 0.05; **P < 0.01.

Treatment with bafilomycin or apocynin led to an increase in bacterial viability (approximately twice that in the controls). Bafilomycin plus apocynin increased viability by ∼4 times that in the control, and DPI increased viability by ∼5 times (**Fig. 8A**). Figure 8B—D shows the results of experiments performed to confirm that the assay used to detect the viability of bacteria inside of neutrophils was performing properly. Figure 8B shows that bafilomycin, apocynin, and DPI did not significantly affect the number of bacteria in culture. Figure 8C indicates that >99.9% of nonengulfed extracellular bacteria was killed (<0.01% of the bacteria survived) by 100 *µ*g/ml gentamicin; the viability of the residual, surviving bacteria was much lower than that of the bacteria inside of the neutrophils. On the other hand, the number of engulfed bacteria was not reduced significantly by treatment with 100 ng/ml gentamicin; this concentration was achieved by being diluted through the process of washing the neutrophil pellets and destroying the cell membrane of the neutrophils (Fig. 8D). Figure 8E shows that neither gentamicin nor bafilomycin A1 affected O_2_^−^ release from neutrophils stimulated with drug-resistant *E. coli*. However, treatment with apocynin led to a partial reduction in the amount of O_2_^−^ released (to ∼50% of that in the control), and DPI treatment led to a marked reduction (to ∼20% of that in the control). We next examined the effect of bafilomycin A1, apocynin, and DPI on autophagy in neutrophils stimulated with drug-resistant *E. coli* in the presence of 1% IgG-free serum and 1 mg/ml IVIG by immunoblot analysis of time-dependent LC3B conversion (Fig. 8F). In nontreated neutrophils stimulated with bacteria, the LC3B-II/I ratio peaked at 3 hours of incubation (lane 4) and decreased thereafter (lanes 5 and 6). These results indicated autophagy induction in bacteria-stimulated neutrophils. Compared with that in nontreated samples, the LC3B-II/I ratio in the apocynin-treated samples decreased at 2–5 hours (lanes 9 and 10). Furthermore, the ratio in DPI-treated samples decreased markedly at the same time-points (lanes 13 and 14). These results suggest that apocynin and DPI, which partially or strongly inhibit the release of O_2_^−^ from neutrophils, respectively, also inhibit autophagy in neutrophils. These data suggest the possibility that autophagy is partially suppressed by apocynin and almost completely suppressed by DPI. These results are consistent with previously published data [17] and indicate that phagocytosis-mediated autophagy is dependent on O_2_^−^ generation by NADPH oxidase. Bafilomycin inhibits fusion of lysosomes with autophagosomes, and as a result, autophagy-related proteins, such as LC3 are not degraded and accumulate in cytoplasm [18]. The LC3B-II/I ratio in the bafilomycin A1-treated samples at 2 hours was almost the same as that in nontreated samples (lane 7). However, at 5 hours, the ratio in the bafilomycin A1-treated samples clearly increased compared with that in nontreated samples (lane 8). These results indicate that autophagosomes accumulate in the cytosol, as bafilomycin A1 prevents them from fusing with lysosomes. This indicates that bafilomycin A1 inhibits the late phase of autophagy. The same effect was observed in the presence of bafilomycin and apocynin (lanes 11 and 12). These results also suggest that apocynin partially inhibits autophagy by blocking the production of O_2_^−^.

## DISCUSSION

The results of the present study showed that IVIG promotes neutrophil-mediated bactericidal activity against ESBL-producing *E. coli* and MDRP. IVIG treatment induced neutrophils to kill and phagocytose bacteria, release O_2_^−^ and MPO, and form NETs. Complement proteins and antibodies cooperate to lyse bacteria (immunobacteriolysis) [19]; however, Fig. 1 shows that the addition of IVIG and IgG-free serum in the absence of neutrophils resulted in limited bacterial killing. This suggests that immunobacteriolysis is not effective if there is a lack of complement and antibodies at the infection site. Thus, phagocytosis and neutrophil-mediated bactericidal activity play important roles in host defense when large numbers of bacteria are present. However, Fig. 1 also shows that IgG-free serum alone had little effect on the number of viable bacteria, even in the presence of neutrophils. This indicates that phagocytosis and neutrophil-mediated bactericidal activity cannot operate effectively if the total amount of IgG (or pathogenic organism-specific IgG) in the tissues and/or blood is very low; thus, such conditions may contribute to severe infection. The results of the present study provide some mechanistic insights into the antibacterial activity of IVIG when used to treat severe infections caused by multidrug-resistant bacteria. The recent increase in antibiotic drug-resistant strains means that treatment of nosocomial infections continues to be a significant clinical challenge. IVIG is an important therapeutic option, as the antibacterial activity of neutrophils is largely independent of the mechanisms that underlie drug resistance. At present, however, little is known about how IVIG mediates bactericidal activity against specific bacterial strains. A thorough understanding of the relationship between IVIG efficacy and the antigenic profile of specific pathogens is critical if we are to optimize IVIG therapy.

Here, we found that IVIG increased the level of phagocytosis-mediated autophagy in neutrophils stimulated with drug-resistant *E. coli* and *P. aeruginosa*. Recent studies suggest that autophagy is more selective than originally thought. The term xenophagy refers to the selective degradation of intracellular pathogens, whereas macroautophagy (historically referred to as “autophagy”) refers to the degradation of intracellular organelles and proteins. TEM analyses indicated that the autophagosomes formed in neutrophils in response to bacterial stimulation contained bacteria and organelles (Fig. 7C), or the autophagosomes formed in neutrophil contained bacteria only and organelles only; therefore, the results of the present study suggest that macroautophagy and xenophagy can operate side by side. The finding that autophagy targets bacteria and organelles is novel and unexpected. The colocalization of bacteria and organelles within the autophagosomes may suggest that a nonselective bulk degradation process is operating; however, it is also possible that the contents of these autophagosomes were selectively delivered by a specific autophagic system, which confers an advantage on the neutrophil. Autophagy is thought to contribute directly and indirectly to host defense. During xenophagy, bacteria that have escaped from phagosomes are engulfed by the autophagosome membrane and degraded via lysosomes. Although most studies of bacterial autophagy focused on the clearance of intracellular pathogens, a recent study showed that autophagy plays an essential role in the clearance of *P. aeruginosa*, a gram-negative extracellular pathogen, by macrophages [20]. Indeed, the results of the present study indicate that IVIG-enhanced autophagy may play a role in neutrophil-mediated bactericidal activity against multidrug-resistant bacteria.

Recent evidence suggests that ROS are required to activate antibacterial autophagy [17]. In addition, it has been reported that the NET formation requires autophagy and ROS generation [21]. The present study also indicates that NET formation occurs after induction of autophagy. NADPH oxidase, which generates ROS, is assembled on the phagosome membrane in response to appropriate signaling via Fc*γ*R and TLR [22]. Sanjuan and coauthors [12, 23, 24] reported a model of LAP in which TLR-mediated recruitment of LC3 to the phagosome results in accelerated maturation, thereby reducing the possibility that microorganisms subvert the phagosomes. These findings show that TLR signaling may play a central role in the induction of autophagy. The current study showed that LC3B conversion in neutrophils in response to bacterial stimulation was suppressed by the NADPH oxidase inhibitors, apocynin and DPI (Fig. 8E and F, lanes 9, 10, 13, and 14), suggesting that NADPH oxidase is involved in activating the autophagic machinery. In addition, we did not detect IVIG-induced autophagy (as assessed by immunoblot analysis of LC3B conversion; Fig. 5A). During LAP, maturing phagosomes recruit LC3 along with NADPH oxidase components and TLRs. Therefore, it is considered that these proteins are colocalized in the phagosomes within a few minutes after phagocytosis. In contrast, the immunoblotting data (Fig. 8F) suggest that autophagy is not induced until ∼2 hours after phagocytosis. As we have not observed the colocalization of LC3B with bacteria in the confocal immunofluorescence microscopy until at least 1 hour (data not shown), we have considered that IVIG enhance not LAP pathway but xenophagy. Moreover, we showed that inhibiting autophagy with NADPH oxidase inhibitors reduces neutrophil-mediated bactericidal activity (Fig. 8A), suggesting that the autophagic machinery may contribute, directly or indirectly, to the neutrophil-mediated killing of bacteria. We also showed that directly inhibiting the late phase of autophagy with bafilomycin A1 reduces the bactericidal activity of neutrophils without affecting O_2_^−^ release. In conditions under which O_2_^−^ release was reduced by apocynin, bafilomycin A1 further decreased bactericidal activity of neutrophils to levels comparable with those achieved by DPI, which strongly inhibited O_2_^−^ release and autophagy (Fig. 8A and E). These findings suggest that the autophagic machinery may play a role in the bactericidal activity of neutrophils independently of O_2_^−^ production. If O_2_^−^ production is reduced, then the autophagic machinery may play a complementary role in antibacterial activity.

Ozone production kills bacteria in patients with CGD, which is characterized by a defect in ROS production as a result of a congenital defect in NADPH oxidase [25]. Patients with CGD often experience life-threatening, recurrent bacterial and fungal infections. The bactericidal activity of neutrophils is an important component of the host primary immune defense system; thus, it is not unreasonable to postulate the existence of several, perhaps parallel, antibacterial mechanisms. In fact, a previous study demonstrates that defective ATG recruitment plays a role in the development of invasive fungal infections of monocytes in CGD patients [26]. Thus, autophagy does play a role in microbicidal responses and may be one such mechanism in neutrophils. Huang et al. [17] reported that the engagement of TLRs or Fc*γ*Rs (both of which activate NADPH oxidase) during phagocytosis induces recruitment of the ATG, LC3, to phagosomes. Thus, IVIG may enhance neutrophil autophagy via Fc*γ*Rs, particularly in immunocompromised hosts, such as transplant recipients or patients with CGD. We are currently conducting experiments that use neutrophils obtained from patients after stem cell transplantation.

In summary, the present study showed that IVIG promotes neutrophil bactericidal activity through phagocytosis of multidrug-resistant and drug-sensitive strains of *E. coli* and *P. aeruginosa*. We also showed that autophagy and NET formation may increase this activity. The identification of the mechanism(s) by which IVIG modulates the bactericidal activity (and other specific functions) of neutrophils is critical if we are to further develop IVIG for use in appropriate clinical settings.

## AUTHORSHIP

H.I. and H.M. performed most of the experimental work and data analysis and made an equal contribution to data interpretation and manuscript preparation. N.K., S.Y., and T.H. performed some of the experimental work and data analysis. H.T., Y.K., T.K., K.Y., M.S., and A.T.-K. contributed to experimental design and data interpretation. S.A. contributed to the experimental design, data interpretation, and preparation of the manuscript.

## Supplementary Material

Supplemental Data

## Abbreviations

ATG5: autophagy protein 5
CGD: chronic granulomatous disease
DPI: diphenyleneiodonium
ESBL: extended-spectrum β lactamase
IVIG: i.v. Ig
Kaketsuken: Chemo-Sero-Therapeutic Research Institute
LAP: LC3-associated phagocytosis
MDRP: multidrug-resistant *Pseudomonas aeruginosa*
MPO: myeloperoxidase
NET: neutrophil extracellular trap
O_2_^−^: superoxide
OsO_4_: osmium tetroxide
RIPA: radioimmunoprecipitation assay
ROS: reactive oxygen species
SEM: scanning electron microscopy
SOD: superoxide dismutase
TEM: transmission electron microscopy
