# Supplementary material for: Enhancement of neutrophil autophagy by an IVIG preparation against multidrug-resistant bacteria as well as drug-sensitive strains
Source: J Leukoc Biol. 2015 Apr 23;98(1):107–17. doi: 10.1189/jlb.4A0813-422RRR (PMC4467167; doi:10.1189/jlb.4A0813-422RRR)
Supplement: Supplemental Data [file supp_98_1_107__index.html]

Enhancement of neutrophil autophagy by an IVIG preparation against multidrug-resistant bacteria as well as drug-sensitive strains — Enhancement of neutrophil autophagy by an IVIG preparation against multidrug-resistant bacteria as well as drug-sensitive strains — Supplemental Data 

# Enhancement of neutrophil autophagy by an IVIG preparation against multidrug-resistant bacteria as well as drug-sensitive strains

## Supplemental Data

**Files in this Data Supplement:**

- Supplemental Data
- Supplemental Data
- Supplemental Data
