# Supplementary material for: Enhancement of neutrophil autophagy by an IVIG preparation against multidrug-resistant bacteria as well as drug-sensitive strains
Source: J Leukoc Biol. 2015 Apr 23;98(1):107–17. doi: 10.1189/jlb.4A0813-422RRR (PMC4467167; doi:10.1189/jlb.4A0813-422RRR)
Supplement: Supplemental Data [file supp_jlb.4A0813-422RRR_Supplemental_Data.docx]

**Supplementary Table 1**

Results of the antibiotic susceptibility tests of the bacterial strains used in this study.

Strain no.1-4; Drug-sensitive strains of *E. coli*, 5-12; (ESBL)-producing strains of *E. coli*, 13-19; Drug-sensitive strains of *P. aeruginosa*, 20-28; *multi-drug-resistant P. aeruginosa* (MDRP).

S： Sensitive, R： Resistance, I： Intermediate, NT： Not tested.

ABPC: ampicillin, PIPC: piperacillin, TAZ/PIPC: tazobactam/piperacillin, CPDX: cefpodoxime proxetil, CEZ: cefazolin, CMZ: cefmetazole, FMOX: flomoxef, CAZ: ceftazidime, CTX: cefotaxime, CPZ: cefoperazone, CFPM: cefepime, AZT: aztreonam, IPM: imipenem, LVFX: levofloxacin, AMK: amikacin, GM: gentamicin, ST: sulfamethoxazole-trimethoprim, MINO: minocycline, TOB: tobramycin, CPFX: ciprofloxacin, CZOP: cefozopran, MEPM: meropenem, AMPC/CV: amoxicillin/clavulanic acid.

**Supplementary Figure 1**

Effect of IVIG on NETs formation by human neutrophils.

The Effect of IVIG on NETs formation in the presence of drug-resistant *E. coli*. The magnified images are representative of the entire sample. “Control”, sample contains neutrophils alone; “*E. coli*”, sample contains both neutrophils and *E. coli*; “*E. coli* + IVIG”, sample contains neutrophils, *E. coli*, and IVIG; “*E. coli* + IVIG + DNase”, sample contains neutrophils, *E. coli*, IVIG, and DNase. Each sample contained 1% IgG-free serum. The right panel shows a magnified image of the rectangle shown on the “*E. coli* + IVIG” sample. The arrow indicates *E. coli* trapped by NETs. Data are representative of three independent experiments using different bacterial strains respectively.
