# Supplementary figures and images for: Enhancement of neutrophil autophagy by an IVIG preparation against multidrug-resistant bacteria as well as drug-sensitive strains
Source: J Leukoc Biol. 2015 Apr 23;98(1):107–17. doi: 10.1189/jlb.4A0813-422RRR (PMC4467167; doi:10.1189/jlb.4A0813-422RRR)

## Slide 1
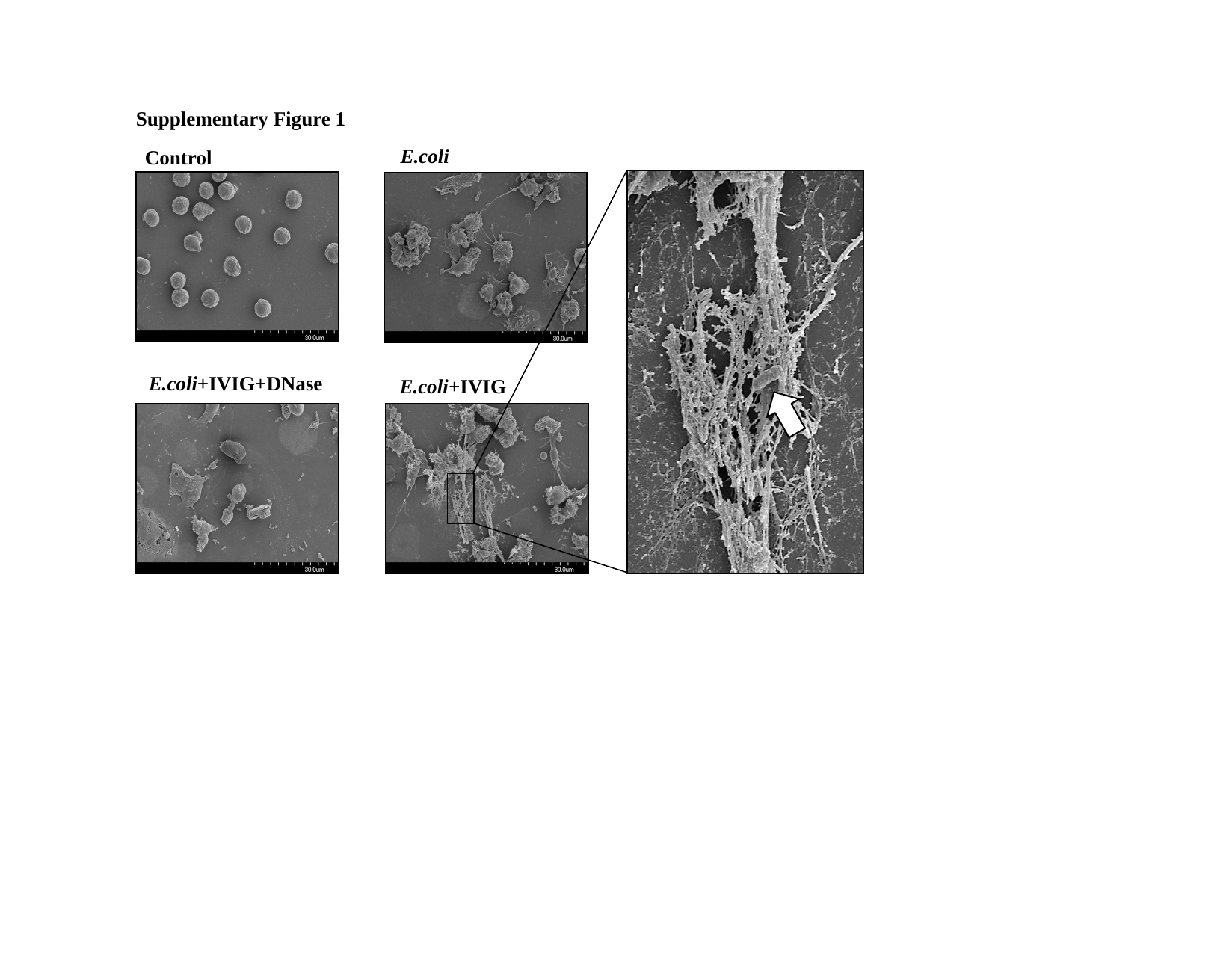

Supplementary Figure 1
E.coli
Control
E.coli+IVIG+DNase
E.coli+IVIG

Supplement: Supplemental Data [file supp_jlb.4A0813-422RRR_Supplemental_Figure.pptx]
