# Supplementary material for: Enhancement of neutrophil autophagy by an IVIG preparation against multidrug-resistant bacteria as well as drug-sensitive strains
Source: J Leukoc Biol. 2015 Apr 23;98(1):107–17. doi: 10.1189/jlb.4A0813-422RRR (PMC4467167; doi:10.1189/jlb.4A0813-422RRR)
Supplement: Supplemental Data [file supp_jlb.4A0813-422RRR_Supplemental_Table.pptx]

## Slide 1
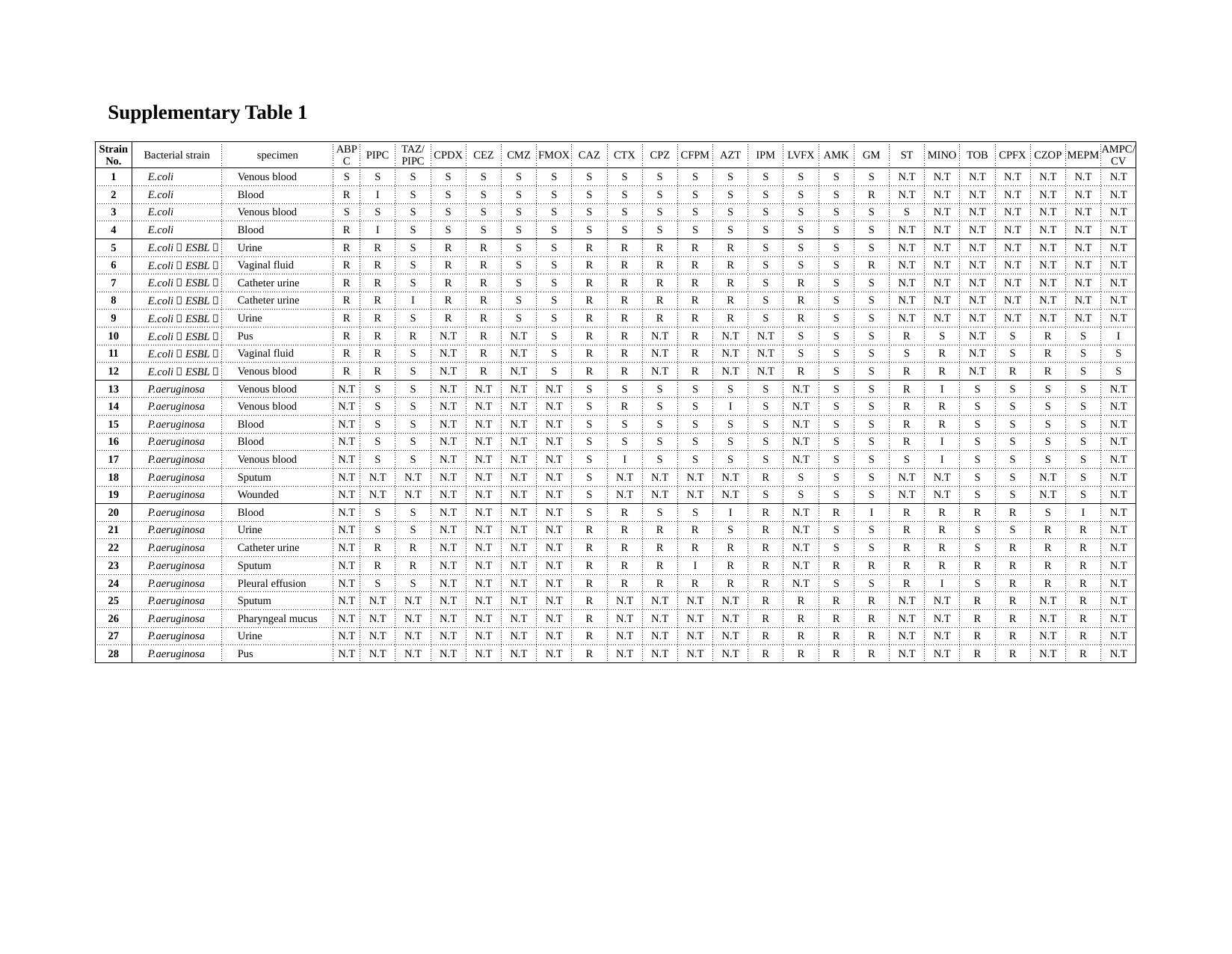

Supplementary Table 1
| Strain No. | Bacterial strain | specimen | ABPC | PIPC | TAZ/ PIPC | CPDX | CEZ | CMZ | FMOX | CAZ | CTX | CPZ | CFPM | AZT | IPM | LVFX | AMK | GM | ST | MINO | TOB | CPFX | CZOP | MEPM | AMPC/CV |
| --- | --- | --- | --- | --- | --- | --- | --- | --- | --- | --- | --- | --- | --- | --- | --- | --- | --- | --- | --- | --- | --- | --- | --- | --- | --- |
| 1 | E.coli | Venous blood | S | S | S | S | S | S | S | S | S | S | S | S | S | S | S | S | N.T | N.T | N.T | N.T | N.T | N.T | N.T |
| 2 | E.coli | Blood | R | I | S | S | S | S | S | S | S | S | S | S | S | S | S | R | N.T | N.T | N.T | N.T | N.T | N.T | N.T |
| 3 | E.coli | Venous blood | S | S | S | S | S | S | S | S | S | S | S | S | S | S | S | S | S | N.T | N.T | N.T | N.T | N.T | N.T |
| 4 | E.coli | Blood | R | I | S | S | S | S | S | S | S | S | S | S | S | S | S | S | N.T | N.T | N.T | N.T | N.T | N.T | N.T |
| 5 | E.coli（ESBL） | Urine | R | R | S | R | R | S | S | R | R | R | R | R | S | S | S | S | N.T | N.T | N.T | N.T | N.T | N.T | N.T |
| 6 | E.coli（ESBL） | Vaginal fluid | R | R | S | R | R | S | S | R | R | R | R | R | S | S | S | R | N.T | N.T | N.T | N.T | N.T | N.T | N.T |
| 7 | E.coli（ESBL） | Catheter urine | R | R | S | R | R | S | S | R | R | R | R | R | S | R | S | S | N.T | N.T | N.T | N.T | N.T | N.T | N.T |
| 8 | E.coli（ESBL） | Catheter urine | R | R | I | R | R | S | S | R | R | R | R | R | S | R | S | S | N.T | N.T | N.T | N.T | N.T | N.T | N.T |
| 9 | E.coli（ESBL） | Urine | R | R | S | R | R | S | S | R | R | R | R | R | S | R | S | S | N.T | N.T | N.T | N.T | N.T | N.T | N.T |
| 10 | E.coli（ESBL） | Pus | R | R | R | N.T | R | N.T | S | R | R | N.T | R | N.T | N.T | S | S | S | R | S | N.T | S | R | S | I |
| 11 | E.coli（ESBL） | Vaginal fluid | R | R | S | N.T | R | N.T | S | R | R | N.T | R | N.T | N.T | S | S | S | S | R | N.T | S | R | S | S |
| 12 | E.coli（ESBL） | Venous blood | R | R | S | N.T | R | N.T | S | R | R | N.T | R | N.T | N.T | R | S | S | R | R | N.T | R | R | S | S |
| 13 | P.aeruginosa | Venous blood | N.T | S | S | N.T | N.T | N.T | N.T | S | S | S | S | S | S | N.T | S | S | R | I | S | S | S | S | N.T |
| 14 | P.aeruginosa | Venous blood | N.T | S | S | N.T | N.T | N.T | N.T | S | R | S | S | I | S | N.T | S | S | R | R | S | S | S | S | N.T |
| 15 | P.aeruginosa | Blood | N.T | S | S | N.T | N.T | N.T | N.T | S | S | S | S | S | S | N.T | S | S | R | R | S | S | S | S | N.T |
| 16 | P.aeruginosa | Blood | N.T | S | S | N.T | N.T | N.T | N.T | S | S | S | S | S | S | N.T | S | S | R | I | S | S | S | S | N.T |
| 17 | P.aeruginosa | Venous blood | N.T | S | S | N.T | N.T | N.T | N.T | S | I | S | S | S | S | N.T | S | S | S | I | S | S | S | S | N.T |
| 18 | P.aeruginosa | Sputum | N.T | N.T | N.T | N.T | N.T | N.T | N.T | S | N.T | N.T | N.T | N.T | R | S | S | S | N.T | N.T | S | S | N.T | S | N.T |
| 19 | P.aeruginosa | Wounded | N.T | N.T | N.T | N.T | N.T | N.T | N.T | S | N.T | N.T | N.T | N.T | S | S | S | S | N.T | N.T | S | S | N.T | S | N.T |
| 20 | P.aeruginosa | Blood | N.T | S | S | N.T | N.T | N.T | N.T | S | R | S | S | I | R | N.T | R | I | R | R | R | R | S | I | N.T |
| 21 | P.aeruginosa | Urine | N.T | S | S | N.T | N.T | N.T | N.T | R | R | R | R | S | R | N.T | S | S | R | R | S | S | R | R | N.T |
| 22 | P.aeruginosa | Catheter urine | N.T | R | R | N.T | N.T | N.T | N.T | R | R | R | R | R | R | N.T | S | S | R | R | S | R | R | R | N.T |
| 23 | P.aeruginosa | Sputum | N.T | R | R | N.T | N.T | N.T | N.T | R | R | R | I | R | R | N.T | R | R | R | R | R | R | R | R | N.T |
| 24 | P.aeruginosa | Pleural effusion | N.T | S | S | N.T | N.T | N.T | N.T | R | R | R | R | R | R | N.T | S | S | R | I | S | R | R | R | N.T |
| 25 | P.aeruginosa | Sputum | N.T | N.T | N.T | N.T | N.T | N.T | N.T | R | N.T | N.T | N.T | N.T | R | R | R | R | N.T | N.T | R | R | N.T | R | N.T |
| 26 | P.aeruginosa | Pharyngeal mucus | N.T | N.T | N.T | N.T | N.T | N.T | N.T | R | N.T | N.T | N.T | N.T | R | R | R | R | N.T | N.T | R | R | N.T | R | N.T |
| 27 | P.aeruginosa | Urine | N.T | N.T | N.T | N.T | N.T | N.T | N.T | R | N.T | N.T | N.T | N.T | R | R | R | R | N.T | N.T | R | R | N.T | R | N.T |
| 28 | P.aeruginosa | Pus | N.T | N.T | N.T | N.T | N.T | N.T | N.T | R | N.T | N.T | N.T | N.T | R | R | R | R | N.T | N.T | R | R | N.T | R | N.T |
